# Development of *Phaleria macrocarpa* (Scheff.) Boerl Fruits Using Response Surface Methodology Focused on Phenolics, Flavonoids and Antioxidant Properties

**DOI:** 10.3390/molecules23040724

**Published:** 2018-03-22

**Authors:** Khurul Ain Mohamed Mahzir, Siti Salwa Abd Gani, Uswatun Hasanah Zaidan, Mohd Izuan Effendi Halmi

**Affiliations:** 1 Lapsah, IPPH, Universiti Putra Malaysia, Putra Infoport, 43400 UPM Serdang, Selangor, Malaysia; ayyeenaruto@gmail.com; 2 Department of Agriculture Technology, Faculty of Agriculture, Universiti Putra Malaysia, 43400 UPM Serdang, Selangor, Malaysia; 3 Department of Biochemistry, Faculty of Biotechnology and Biomolecular Science, Universiti Putra Malaysia, 43400 UPM Serdang, Selangor, Malaysia; uswatun@upm.edu.my; 4 Department of Land Management, Faculty of Agriculture, Universiti Putra Malaysia, 43400 UPM Serdang, Selangor, Malaysia; m_izuaneffendi@upm.edu.my

**Keywords:** *Phaleria macrocarpa*, extraction, free radical scavenging activity (DPPH), ferric ion reducing power assay (FRAP), total phenolic content (TPC), total flavonoid content (TFC), response surface methodology (RSM)

## Abstract

In this study, the optimal conditions for the extraction of antioxidants from the Buah Mahkota Dewa fruit (*Phaleria macrocarpa)* was determined by using Response Surface Methodology (RSM). The optimisation was applied using a Central Composite Design (CCD) to investigate the effect of three independent variables, namely extraction temperature (°C), extraction time (minutes) and extraction solvent to-feed ratio (% *v*/*v*) on four responses: free radical scavenging activity (DPPH), ferric ion reducing power assay (FRAP), total phenolic content (TPC) and total flavonoid content (TFC). The optimal conditions for the antioxidants extraction were found to be 64 °C extraction temperature, 66 min extraction time and 75% *v*/*v* solvent to-feed ratio giving the highest percentage yields of DPPH, FRAP, TPC and TFC of 86.85%, 7.47%, 292.86 mg/g and 3.22 mg/g, respectively. Moreover, the data were subjected to Response Surface Methodology (RSM) and the results showed that the polynomial equations for all models were significant, did not show lack of fit, and presented adjusted determination coefficients (*R*^2^) above 99%, proving that the yield of phenolic, flavonoid and antioxidants activities obtained experimentally were close to the predicted values and the suitability of the model employed in RSM to optimise the extraction conditions. Hence, in this study, the fruit from *P. macrocarpa* could be considered to have strong antioxidant ability and can be used in various cosmeceutical or medicinal applications.

## 1. Introduction

Natural and healthy lifestyles are gaining the attention of people worldwide since 2000. There is a thriving interest in natural bioactive compounds in the human diet or for application in natural medicine [1]. The medicinal properties of these natural bioactive compounds have been investigated scientifically all over the world due to their abundance of antioxidant properties, no side effects and low cost [2]. Besides, traditional medicinal uses of plants are topics for research because medicines from plants have good advantages such as low toxicity if used in the right dose, low cost, fewer side effects and being easy to obtain. One of such plants is *Phaleria macrocarpa* (Scheff.) Boerl, which belongs to the Thymelaeceae family. It is variously known as the ‘Crown of God’, ‘Mahkota Dewa’ and ‘Pau’. It is a native plant from the tropical areas of the Papua Island, Indonesia and grows up to 5–18 m tall. It can be found up to 1200 m above the sea level [3]. It is a complete tree with stem, leaves, flowers and fruits. The leaves are green and acuminate with length and diameter range from 7–10 cm and 3–5 cm approximately. The flowers can make clusters of 2–4 with colours ranging from green to maroon. The pit is round, white and poisonous [4]. The fruits are green when they are not ripened and become red when fully ripened [5] while fruits have 1 to 2 seeds which are brown, egg-shaped and anatropous.

Traditionally, the locals have used *P. macrocarpa* as a herbal drink either on its own or mixed with other medicinal plants to treat illnesses such as cancer, hypertension and diabetes mellitus [6]. The biological and pharmacological activities of the stem, leaves, fruit and seed parts have been examined by several researchers [7]. The fruits can cure high blood pressure, gout, skin disease, liver, cancer and diabetes [3]. The stems are used to treat bone cancer; the egg shells of the seeds are used as antidote for the breast cancer, cervix cancer, lung disease and heart disease, while the leaves contain constituents that treat impotence, allergies, blood disease and tumours [8]. Regarding on the beneficial properties of this plant, it has been reported that *P. macrocarpa* has medicinal activities such as anti-tumour, anti-hyperglycemia, anti-inflammation, anti-diarrheal, anti-oxidant, anti-viral, anti-bacterial, anti-fungal and vasodilator effect [9].

The characterisation of bioactive compounds in *P. macrocarpa* seed extracts using GC-MS found methyl stearate, oleic acid, methyl oleate, linoleic acid, methyl linolenate and palmitic acid in a hexane extract, while the chloroform extract showed the presence of methyl myristate, palmitic acid, methyl oleate, methyl linoleate, *Z*-oleic acid and *Z*-9,17-octadecadienal [10]. Moreover, the phytochemical studies on the fruits of *P. macrocarpa* yielded 2,6,4′-trihydroxy-4-methoxy-benzophenone and 6,4′-dihydroxy-4-methoxybenzophenone-2-*O*-β-d-glucopyranoside in the ethanol extract together with two triterpenoid compounds, 24-methylenecycloartan-3-one and 24-methyl-9,19-cyclolanost-25-en-3-ol, isolated from a chloroform extract. The purification of the *n*-hexane extract of the leaves gave β-sitosterol and stigmasterol [11]. The presence of flavonoids such as kaempferol, naringin, myricetin, rutin and quercetin in the fruit extract of *P. macrocarpa* provides antibacterial activity against pathogenic microorganisms. The bark and fruit in leaves of *P. macrocarpa* contain saponins, alkaloids, poyphenolics, phenols, flavonoids, lignins and tannins. Moreover, there is the presence of major fatty acid components in *P. macrocarpa* seed oil such as 9-octadecanoic acid, linoleic acid, and palmitic acid, followed by a minor amounts stearic acid, methyl tetradecanoate, methyl hexadecanoate, methyl 9,12-octadecanoate, methyl 9-octadecanoate, methyl octadecanoate, methyl *trans*-3-octadecanoate, methyl 11-eicosanoate and methyl nonadecanoate as represented by the GC-MS chromatogram [12].

In order to construct empirical models that are able to find effective statistical relationships between all the variables in an industrial system, Response Surface Methodology (RSM) was introduced by Box and Wilson in 1951 within the context of chemical engineering [13]. It is used to detect the factors that influence a response and their interaction. The advantages of using RSM are empowering the evaluation of the effects of certain process variables and their interaction of response variables, less number of required experiments, more speed, less cost, less laboriousness and less time consumption [14]. There are studies regarding the optimisation of antioxidants, phenolic and flavonoid content by using RSM such as in the extraction of rambutan peel extract [15], defatted Dabai parts [16] and *Annona crassiflora* Mart. (Araticum) [17]. In this research, a Central Composite Design (CCD) was applied to determine the optimum conditions for extraction due to the advantages of lower number of required experiments, chronological investigation and reasonable information of lack of response surface methodology. Hence, the extraction was carried out by using reflux apparatus by focusing on the effect of three independent variables which are the extraction temperature (°C), extraction time (minutes) and solvent to-feed ratio (% *v*/*v*) on four responses: free radical scavenging activity (DPPH), ferric ion reducing power assay (FRAP), phenolic and flavonoid content. These three factors were chosen based on the optimization of oil yield in *P. macrocarpa* seed using RSM and the composition of the fatty acid constituents [12]. However, in the determination of antioxidant properties by using Response Surface Methodology (RSM), there is still a lack of research. Hence, the main objectives of this research are to study the optimised temperature, time and solvent ratio extraction conditions in order to obtain the highest percentage yield in the antioxidant properties of *P. macrocarpa* fruit extract by using RSM.

## 2. Results and Discussion

### 2.1. Model Fitting

Twenty experiments were carried out using different combinations of the independent variables by using Response Surface Methodology and a Central Composite Design (CCD). This was used to identify the relationship between the response functions and process variables. The experimental and predicted values for the responses of DPPH, FRAP, TPC and TFC were acquired. The predicted values were obtained with a model fitting technique by using the software Design Expert 7.0.0 (Trial version, Stat-Ease Inc., Minneapolis, MN, USA). In the present study, according to the sequential model sum of squares, the highest order polynomials were utilised to select the models where the additional coefficients estimates were significant and the models were not aliased. Hence, for all three independent variables and responses, a quadratic polynomial model was selected and fitted well as suggested by the software. 

The independent variables were focusing mainly on three factors to give impact to the results obtained. Solvent extraction is the main commonly used extraction method to recover a wide range of antioxidants and phenolic compounds [18,19]. The effectiveness of extraction is also affected by factors such as storage time, extraction method, solvent types, the pH, extraction temperature, solvent-to-solid ratio, particle size and solvent concentration [20,21].

Furthermore, solvent polarity plays an important role in increasing the solubility of phenolic compounds [17]. The factor of extraction temperature was considered because increasing the extraction temperature improves extraction by increasing the solubility, hence producing better antioxidant activity [22] while use of a higher extraction temperature can cause the bioactive compound to decompose, particularly flavonoids, as concluded from the decrease of antioxidant activity [21]. Moreover, from an industrial point of view, a longer extraction time leads to lower efficiency of equipment utilization, so the extraction time needs to be in a suitable range [23].

Based on Table 1, the result showed that the DPPH, FRAP antioxidant activities, TPC and TFC of crude extracts of *P. macrocarpa* ranged from 83.70% to 86.85%, 7.12–7.47%, 186.22–292.86 mg of gallic acid/g of the extract and 1.92–3.22 mg of quercetin/g of extract, respectively, for the samples treated under different extraction conditions. The maximum DPPH and TPC values were obtained for the extraction time, temperature and solvent ratio of 66.36 min, 70 °C, 80% (*v*/*v*). Meanwhile, the maximum values for FRAP and TFC were obtained for 80 min, 60 °C, 70% (*v*/*v*) respectively. The minimum conditions for DPPH and FRAP antioxidant value were for 120 min, 80 °C and 70% (*v*/*v*). Lastly, the minimum conditions for phenolic and flavonoid content were observed at 100 min, 70 °C and 63.18% (*v*/*v*) and 80 min, 80 °C and 90% (*v*/*v*), respectively.

In order to fit the response function and experimental data to the second-order polynomial, the linearity and quadratic effect of the independent variables, their interactions and regression coefficients on the response variables were evaluated from analysis of variance ANOVA as shown in Table 2. The ANOVA results were calculated based on 94% confidence intervals and this analysis was crucial to determine the best fitted quadratic model for the three independent variables. The regression model was evaluated by using *F* statistics and lack of fit test. Based on the results, it showed that the model is highly significant when the computed *F*-value is greater than the tabulated *F*-value and the probability value is low (*p* < 0.0001), indicating that the individual terms in each response model were significant on the interaction effect. The performance of the models was determined by calculating the determination coefficients *R*^2^, adjusted *R*^2^, predicted *R*^2^, regression (*p*-value), regression (*F*-value), lack of fit (*p*-value), coefficient variation (CV%), lowest PRESS and probability values related to the effect of the three independent variables. Coefficient of determination, *R*^2^ and the significance of lack of fit are the two important aspects that judge the fitness and adequacy of the model obtained. *R*^2^ can be defined as the ratio of the explained variation to the total variation as a measure of the degree of fit [24].

In practice, high determination coefficient (*R*^2^) values (>70%) are reasonable indicators of suitability of regression models to describe the influences of the independent variables on the dependent variables. It indicates that the modelling of experimental data allowed for the generation of useful mathematical equations for general use, within the experimental range tested in this study, to investigate the behaviour of the system under different factor combination [25]. Hence, the coefficient of determination *R*^2^ values for the regression model predicted for DPPH, FRAP, TPC, TFC were 0.9999, 0.9918, 1.000 and 0.9998 respectively, suggesting a good fit. The closer the *R*^2^ value to unity, the better and more significant the empirical model fits the actual data, while the absence of lack of fit (*p* > 0.05) for all the responses also strengthened the accuracy of the models.

Moreover, the calculated adjusted *R*^2^ values for the studied responses variables were higher than 0.80, hence there is a close agreement between the experimental results and the theoretical values predicted by the proposed models. Furthermore, the coefficient variation (CV) is a measure of deviation from the mean value, indicating the reliability of the experiment. Generally, when CV < 10% it shows a better reproducibility [26]. The CV values obtained for DPPH, FRAP, TPC and TFC were 9.65, 0.15, 2.32 and 0.25, respectively, indicating the model is highly reliable and precise.

Other than that, the significance of each coefficient was determined using the *F*-test and *p*-value. The corresponding variables are more significant if the absolute *F* value becomes greater and the *p*-value becomes smaller [27]. The *F*-values for all responses model are greater than the tabulated *F*-value, indicating the adequacy of the models to predict different responses at different extraction conditions. Adequate precision, AP is a comparative measure between the predicted values and mean prediction error. It also measures the signal to noise ratio. A ratio greater than 4 is more desirable which ensures the predicted models are consistent with the independent variables [28].

**Figure 1:**
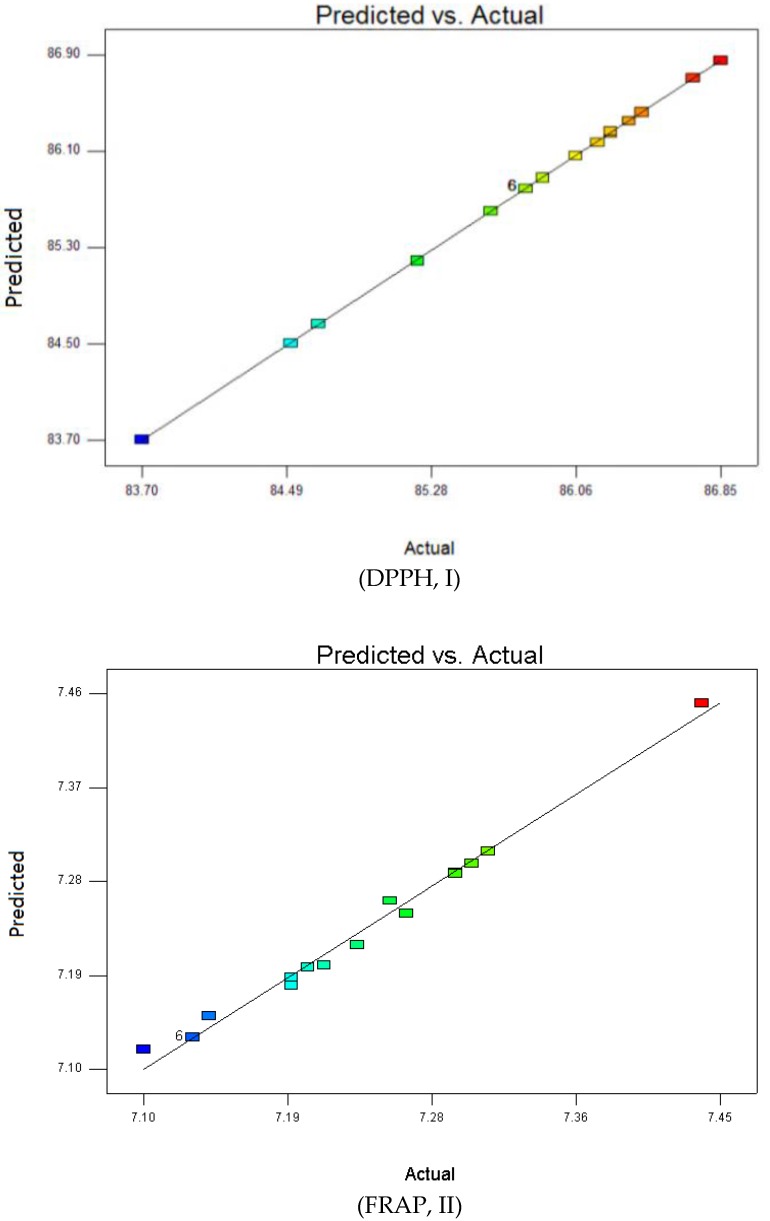

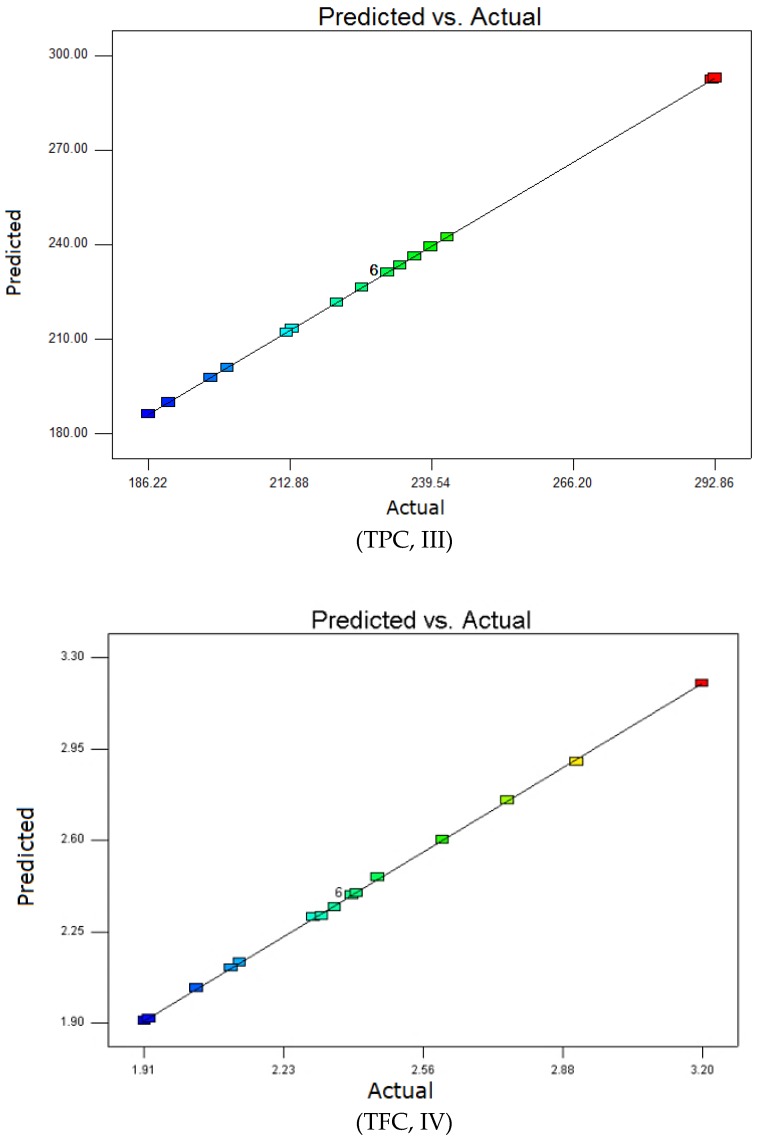
The fitted line plot signifying the closeness between predicted values and experimental values for (DPPH, I), (FRAP, II), (TPC, III) and (TFC, IV).

Lastly, the PRESS values were considered to be minimums to obtain a well-accorded model. It can be concluded from the analysis of ANOVA that any terms from quadratic polynomial coefficients model, large *F*-values and small *p*-values indicated a more significant effect on the respective response variables. The suitability model was investigated based on the results of the fitted-line plot of predicted versus experimental as shown in Figure 1. The diagnostic line plot proved the intimate closeness between predicted and experimental values for all these responses.

### 2.2. Free Radical Scavenging Activity, DPPH

**Figure 2:**
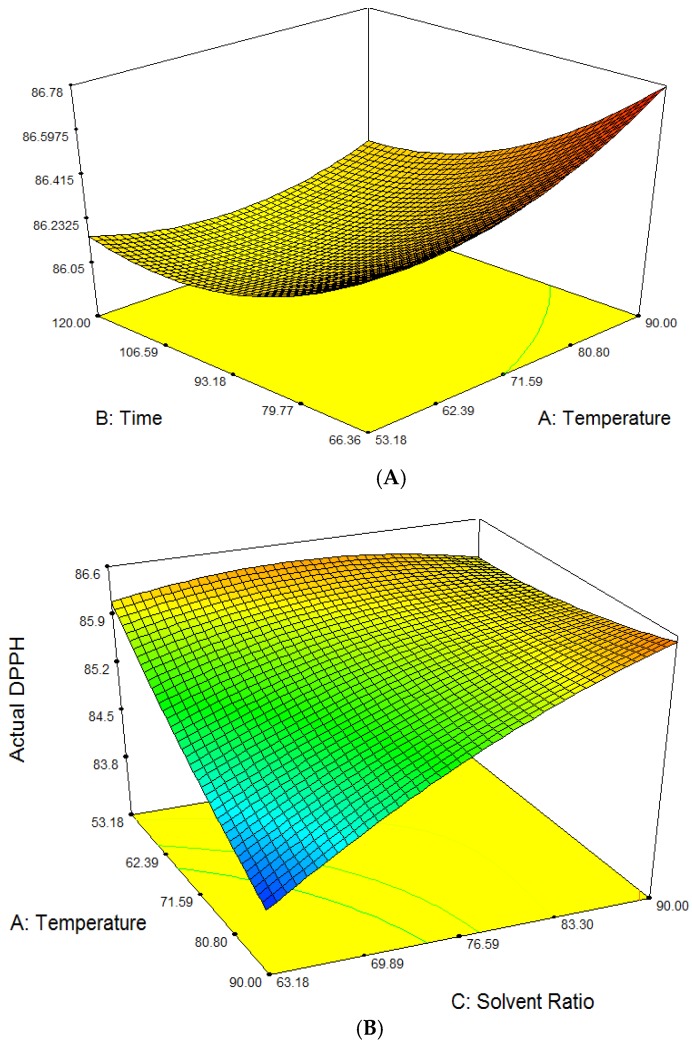

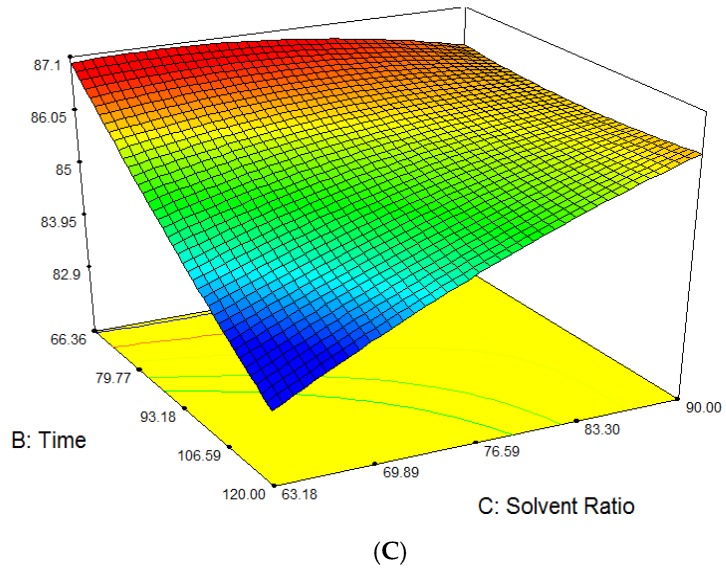
Response surface analysis (3D) on the effect of extraction temperature, ethanol concentration and extraction time on DPPH of *P. macrocarpa* fruit extract. (**A**) DPPH Scavenging Activity (%); (**B**) DPPH Scavenging Activity (%); (**C**) DPPH Scavenging Activity (%).

The amount of extracted antioxidant of free radical scavenging activity (DPPH) content from *Phaleria macrocarpa* fruit extract ranged from 83.70 to 86.70 mg ascorbic acid/g of sample extract. The mean recorded value was 85.76 of the total fruit extracts. The highest DPPH content was reported for experiment No. 1 while the lowest DPPH content was observed in experiment No. 4. The ANOVA showed the model *F*-value of 17783.61 with probability (*p* < 0.0001) which implies that the model is significant and there is only 0.01% chance that this large *F* value could occur due to noise. Free radical scavenging activity was significantly influenced at (*p* < 0.05) by all three linear parameters (*A*, *B*, *C*), interaction parameters (*AB*, *AC*, *BC*) and quadratic parameters (*A*^2^, *B*^2^, *C*^2^). The effect of their variables and their interaction on the responses can be seen in Figure 2A–C. The interactions also gave a significant effect on DPPH for each of the parameter. Ethanol concentration showed the most critical effect on the DPPH of *P. macrocarpa* fruit as it displayed the largest negative regression coefficient for linear effect and interaction effect with extraction temperature. The predicted model of the DPPH scavenging activity was obtained from the following second-order polynomial Equation (1):

*Y_DPPH_* = 85.80 − 0.45 *A* − 1.00 *B* + 0.63 *C* − 0.080 *AB* + 0.69 *AC* + 1.01 *BC* + 0.11 *A*^2^ + 0.15 *B*^2^ − 0.26 *C*^2^ (1)

Effects of the two independent variables on DPPH, when the third variable was fixed in the middle level are shown by three dimensional (3D) response plots in Figure 2. Based on Figure 2A, it is revealed that the predicted response surface of the effect of temperature and time on DPPH was at a constant ethanol concentration of 90% (*v*/*v*). There was a rapid increase in the antioxidant activity of the compound extract as the temperature increased from 60 to 80 °C and time from 80 to 120 min, respectively. 

However, at the constant ethanol concentration of 80% (*v*/*v*), the antioxidant activity of DPPH declined at 100th minute with the temperature at 53.18 °C and 70 °C. According to Pinelo et al. [20] high DPPH scavenging activity could be obtained by increasing the extraction time because it will improve the solubility of solute and increase the extraction coefficients. DPPH also decreased with increasing ethanol concentration and temperature from 63.18–80% (*v*/*v*) and 53.18–70 °C respectively at a constant time of 100 min as shown in Figure 2B.

The increases of DPPH antioxidant activity were only selective when the extraction concentration increased to 80% (*v*/*v*) at lower temperature of 60 °C which indicates solvent polarity plays a vital role in the extraction of the antioxidant compounds. It was safer to use ethanol since it has less toxicity compared to other solvents such as methanol, acetone and others. Therefore, addition of the appropriate amount of water to organic solvents increased its polarity which consequently increased the efficiency of antioxidant compounds extraction.

Furthermore, Figure 2C shows the DPPH decreased when the temperature was kept constant at 70 °C in the range of time of 100–133.64 min and ethanol concentration of 63.18–80% (*v*/*v*). In order to obtain high antioxidant of DPPH, the extraction temperature plays a more critical role in extending the extraction time [29]. In this experiment, it was revealed that as the temperature increased until 80 °C the value of DPPH scavenging activity did not increase significantly as the time increased due to the decomposition of the anti-oxidative compounds which are heat-sensitive.

### 2.3. Ferric Ion Reducing Power Assay, FRAP

**Figure 3:**
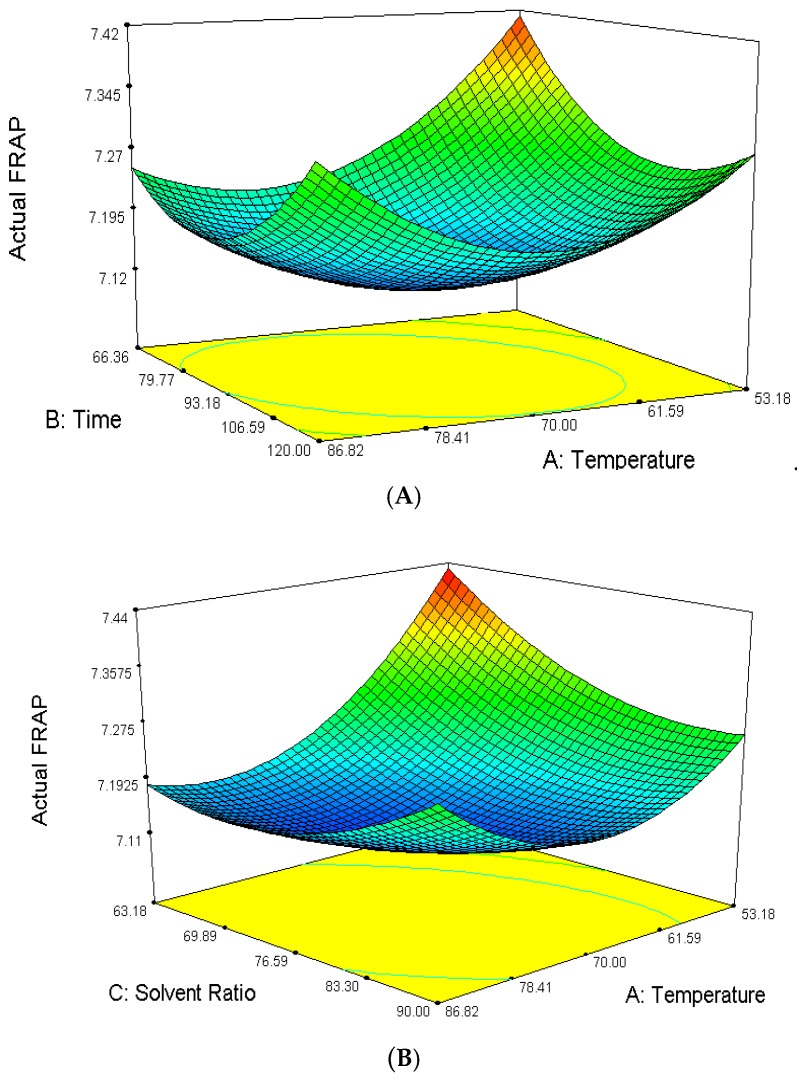

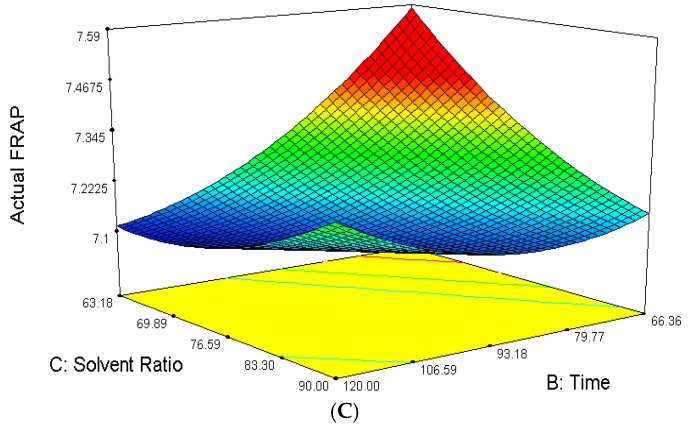
Response surface analysis (3D) on the effect of extraction temperature, ethanol concentration and extraction time on FRAP of *P. macrocarpa* fruit extract. (**A**) Ferric Ion Reducing Power Assay, FRAP (%); (**B**) Ferric Ion Reducing Power Assay, FRAP (%); (**C**) Ferric Ion Reducing Power Assay, FRAP (%).

The amount of extracted antioxidant ferric ion reducing power assay, FRAP content from *Phaleria macrocarpa* fruit extract ranged from 7.12 to 7.47 mg TPTZ/g of the sample extracts. The mean recorded value was 7.21 mg TPTZ/g of the total fruit extracts. The highest FRAP content was reported for experiment No. 1 while the lowest FRAP content was observed in experiment no. 4. The ANOVA showed the model *F*-value of 134.76 with probability (*p* < 0.0001) which implies that the model is significant. There is only a 0.01% chance that this large *F* value could occur due to noise. Ferric ion reducing power assay (FRAP) was significantly influenced at (*p* < 0.05) by all three linear parameters (*A*, *B*, *C*), interaction parameters (*AB*, *AC*, *BC*) and quadratic parameters (*A*^2^, *B*^2^, *C*^2^). The effect of their variables and their interaction on the responses can be seen in Figure 3A–C. The interactions also gave a significant effect on FRAP for each of the parameters. Temperature showed the most critical effect on FRAP of *P. macrocarpa* fruit as it displayed the largest negative regression coefficient for linear effect and interaction effect with the extraction temperature. The predicted model of the FRAP was obtained from the following second-order polynomial Equation (2):

*Y*_FRAP_ = 7.16 − 0.069 *A* − 0.11 *B* − 0.083 *C* + 0.052 *AB* + 0.071 *AC* + 0.14 *BC* + 0.12 *A*^2^ + 0.085 *B*^2^ − 0.056 *C* (2)

Effects of the two independent variables on FRAP, when the third variable was fixed in the middle level are shown by three dimensional (3D) response plots. Based on Figure 3A, it is revealed that the predicted response surface of the effect of temperature and time on FRAP was at a constant ethanol concentration of 90% (*v*/*v*). There was a rapid increase in antioxidant activity of the compound extracted as the temperature increased up to 60–80 °C and time to 80–120 min, respectively. However, at a constant ethanol concentration of 70% (*v*/*v*), the antioxidant activity decreased as the extraction time and extraction temperature increased. This revealed that the thermo-sensitive bioactive antioxidant compounds underwent heat degradation, thus reducing the antioxidant activity Silva et al. [21]. FRAP also showed decreases with the increase of ethanol concentration and temperature from 70–90% (*v*/*v*) and 60–80 °C respectively at a constant time of 80 min as shown in Figure 3B. The decreases of FRAP antioxidant activity indicate that solvent polarity plays a vital role in the extraction of antioxidant compounds. Methanol extract of sea buckthorn seed had higher reducing power than the extract using low polarity chloroform as reported by Negi et al. [30].

Furthermore, Figure 3C shows that FRAP displayed a fluctuation condition when the temperature was kept constant at 70 °C in the range of extraction times of 66.36–133.64 min and extraction ethanol concentration of 63.18–80% (*v*/*v*). In order to obtain a higher FRAP antioxidant activities, polar aqueous solvents dissolve more polar plant polyphenols with higher reducing power at all different extraction times.

### 2.4. Total Phenolic Content, TPC

**Figure 4:**
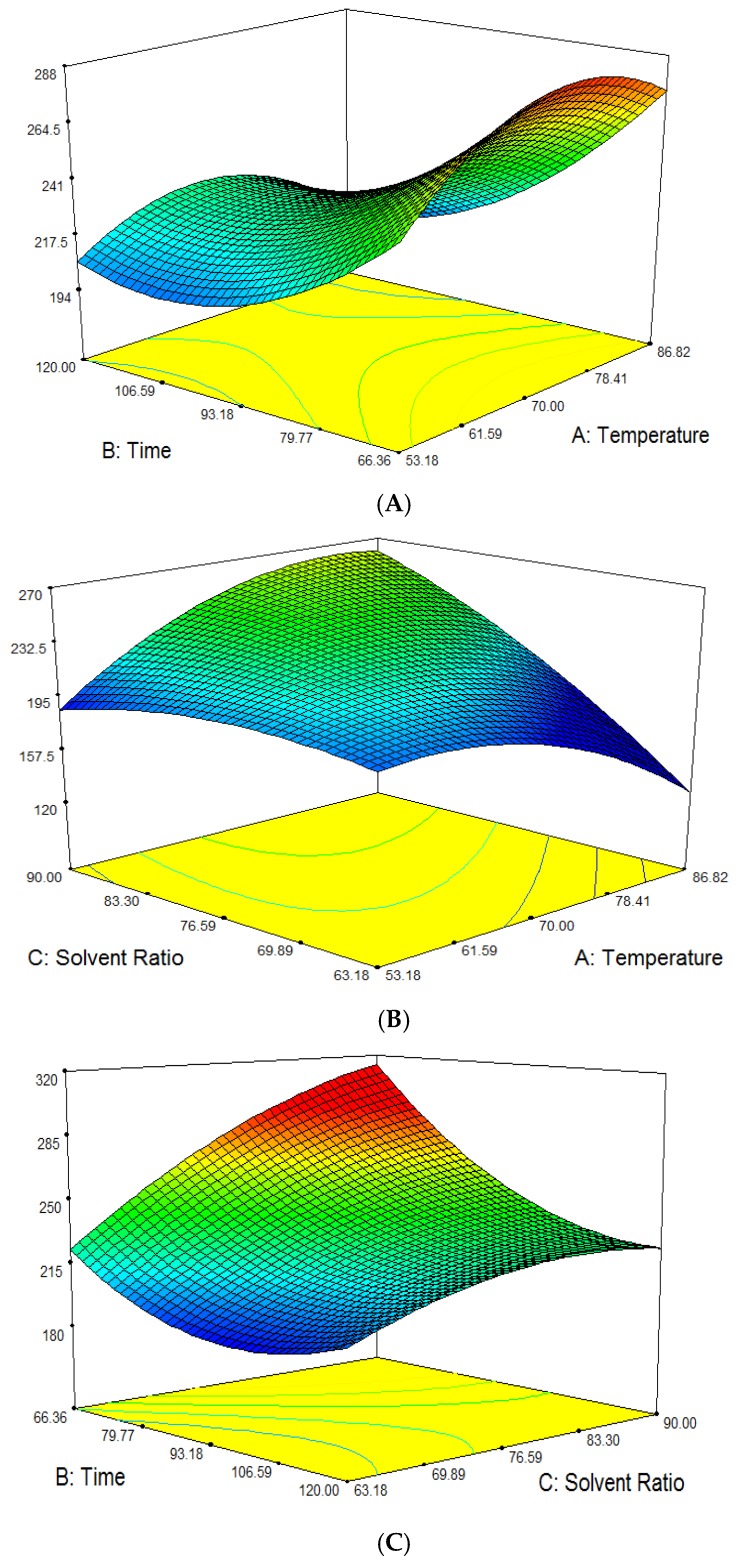
Response surface (3D) on the effect of extraction temperature, ethanol concentration and extraction time on TPC of *P. macrocarpa* fruit extract. (**A**) Total Phenolic Content (mg/g); (**B**) Total Phenolic Content (mg/g); (**C**) Total Phenolic Content (mg/g).

The amount of extracted total phenolic content, TPC, from *P. macrocarpa* fruit extract ranged from 186.22 to 292.86 mg/g gallic acid equivalents (GAE). The mean recorded value was 228.65 mg/g GAE of total leaves extracts. The highest TPC content was reported for experiment No. 11 while the lowest TPC content was observed in experiment No. 13. The ANOVA showed the model *F*-value of 5.585 × 10^7^ with probability (*p* < 0.0001) which implies that the model is significant. There is only a 0.01% chance that this large *F* value could occur due to noise. Total phenolic content, TPC was significantly influenced at (*p* < 0.05) by all three linear parameters (*A*, *B*, *C*), interaction parameters (*AB*, *AC*, *BC*) and quadratic parameters (*A*^2^, *B*^2^, *C*^2^). The effect of their variables and their interaction on the responses can be seen in Figure 4A–C. The interactions also gave a significant effect on TPC for each of the parameters. From Table 2, both the linear and quadratic terms of all parameters were significant with at least *p* < 0.05 on total phenolic content. The interactions also gave a significant effect on TPC for each of the parameters. Ethanol concentrations showed the most critical effect on the TPC of *P. macrocarpa* fruit as it displayed the largest positive regression coefficient for linear and interaction effect with extraction time. The predicted model of the TPC was obtained from the following second-order polynomial Equation (3):

Y_TPC_ = 230.96 − 4.22 *A* − 29.35 *B* + 34.01 *C* − 10.73 AB + 39.58 *AC* − 16.45 *BC* − 30.62 *A*^2^ + 22.19 *B*^2^ − 12.67 *C*^2^ (3)

Based on Figure 4A, it is revealed that the predicted response surface of the effect of temperature and time on TPC was at a constant ethanol concentration of 90% (*v*/*v*). The phenolic compounds gave a higher value for the range of time at 80–120 min and temperature at 60–80 °C. As revealed by [31] higher solubility and diffusion coefficient of polyphenols when the temperature increases favors a higher extraction rate. Moreover, the increase in extraction temperature increased both the solubility of the solutes and their diffusion coefficients, but beyond a certain extent the phenolic compounds could be decomposed. Compound stability could be affected due to chemical and enzymatic degradation or losses by thermal decomposition [29].

Nevertheless, TPC also showed an increase with increasing ethanol concentration and temperature from 70–90% (*v*/*v*) with 60 °C and 80 °C, respectively, at a constant time of 120 min as shown in Figure 4B. Solvent polarity plays an important role in the extraction of antioxidant compounds. Ethanol was chosen because it is safer and less toxic compared to other solvents such as methanol, acetone and others. It was reported that binary-solvent systems demonstrated higher yield of phenolic compounds and flavonoids as compared to monosolvent system comprised of pure solvent or pure water only [19]. In Figure 4C, the highest TPC was attained when the temperature was kept constant at 60 °C, in the range of time of 80–120 min and the ethanol concentration held at 70–90% (*v*/*v*). The phenolic content began to decrease as the extraction duration approached 120 min because it is believed that long extraction times increase the exposure to oxygen that leads to oxidation of antioxidant compounds.

### 2.5. Total Flavonoid Content, TFC

**Figure 5:**
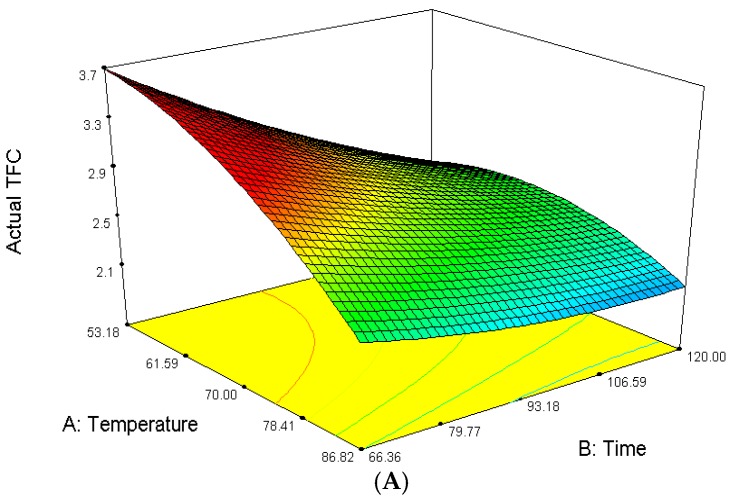

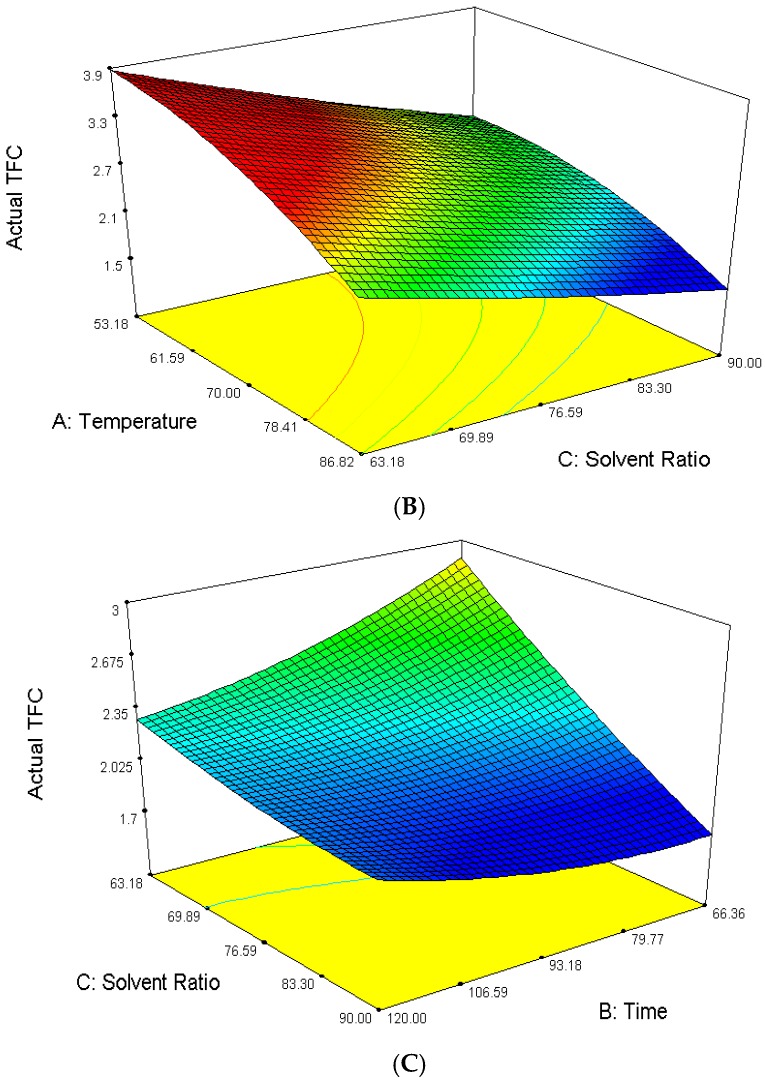
Response surface (3D) on the effect of extraction temperature, ethanol concentration and extraction time on TFC of *P. macrocarpa* fruit. (**A**) Total Flavonoid Content (mg/g); (**B**) Total Flavonoid Content (mg/g); (**C**) Total Flavonoid Content (mg/g).

The amount of extracted total flavonoid content, TFC from *P. macrocarpa* fruit extract ranged from 1.92 to 3.22 mg quercetin/g of sample extract, measured as gallic acid equivalent (GAE). The mean recorded value was 2.39 mg/g GAE of total leaves extracts. The highest TFC content was reported for experiment No. 1 while the lowest TFC content was observed in experiment No. 6. The ANOVA showed the model *F*-value of 5593.88 with probability (*p* < 0.0001) which implies that the model is significant. There is only a 0.01% chance that this large *F* value could occur due to noise. Total flavonoid content, TPC was significantly influenced at (*p* < 0.05) by all three linear parameters (*A*, *B*, *C*), interaction parameters (*AB*, *AC*, *BC*) and quadratic parameters (*A*^2^, *B*^2^, *C*^2^). The effect of their variables and their interaction on the responses can be seen in Figure 5A–C. The interactions also gave a significant effect on TFC for each of the parameters. The predicted model of the TPC was obtained from the following second-order polynomial Equation (4):

Y_TFC_ = 2.51 − 0.41*A* − 0.26*B* − 0.42*C* + 0.28*AB* + 0.10*AC* + 0.23*BC* − 0.28*A*^2^ + 0.091*B*^2^ + 0.049*C*^2^ (4)

Based on Figure 5A, when the ethanol concentration was fixed at 80% (*v*/*v*), the flavonoid content decreased as the temperature increased from 53.18 °C to 70 °C during the range of time from 66.36 min to 133.64 min. At a higher temperature the fluid density will decrease which leads to reduced extraction efficiency [32]. However, at a constant ethanol concentration of 70% (*v*/*v*), with the suitable temperature of 60 °C and time of 80 min, the flavonoid content increased. This may be due to the greater speed of molecular movement at the higher temperature which can cause flavonoids to diffuse more quickly from cells to the extraction solvent [33]. However, an upper limit of temperature must be controlled to prevent the decomposition of particular thermo-sensitive flavonoid compounds [21].

Figure 5B of the 3D response surface plots shows the varying temperatures and ethanol concentrations while the extraction time was kept constant at 80 min. It can be seen that the maximum flavonoid level in the fruit extracts *P. macrocarpa* was attained around the range of temperature and ethanol concentration of 60–80 °C and 70–90% (*v*/*v*), respectively. According to Kumar et al., the optimal extraction yield can be achieved when the polarity of the fluid and its flavonoids are coincident. Moreover, TFC increased with the concentration of ethanol at 55–75% or above 75%. Flavonoid content was decreased in the leaves of *Tabernaemontana heyneana Wall.* Ethanol interacts with the flavonoids possibly through non-covalent interactions and promotes a rapid diffusion into solution [34]. The various concentrations of ethanol used exhibited different effects in changing the fluid polarity. Hence, it produced an effect on the solubility enhancement of the flavonoid [32].

Figure 5C shows the relationship between ethanol concentration and extraction time at a constant temperature of 70 °C. The flavonoid content was observed to be positively influenced by the synergism between the ethanol concentration and extraction time. Fruit extracts of P. macrocarpa displayed a maximum flavonoid content of 3.22 mg of quercetin/g of extract using an ethanol concentration with 70% ethanol when the extraction time was 80 min.

### 2.6. FTIR Analysis

**Figure 6:**
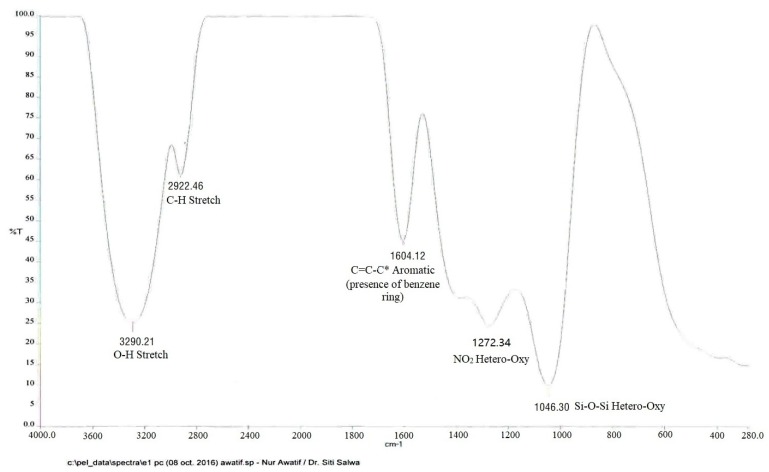
FT-IR of ethanol fruit extracted *P. macrocarpa.*

Fourier Transform Infrared (FTIR) spectroscopy was used to identify the functional groups of the bio-active components that exist in the fruit extract based on the different peak values in the infrared radiation region, as shown in Table 3 and Figure 6. The relative absorption and peak intensity of the fruit components was obtained when the extract was subjected to FTIR and the functional groups of the components were separated based on their peak ratios. The absorption peak at 3295.68 cm^−1^ is due to the hydroxyl group H bonded O-H stretch. It is indicative of the presence of alcohol groups. The O-H bending vibration was broadened by hydrogen bonding to the stretching absorption, but often to a lesser extent. The absorption peak at 2922.92 cm^−1^ is due to the saturated aliphatic group methylene C-H asymmetric (>CH_2_) stretch. The C-H stretch vibrations for methyl and methlyene were the most characteristic in terms of recognizing the compound as an organic compound containing at least one aliphatic fragment or center. Moreover, the aromatic ring stretch was present as the absorption peak 1613.49 cm^−1^ (C=C=C^a^), indicating the presence of benzene rings. The 1273.25 cm^−1^ and 1041.84 cm^−1^ bands indicated the presence of hetero-oxygens bonds for nitrogen-oxygen compounds like organic nitrates and organic siloxanes Si-O-Si, respectively [35].

### 2.7. HPLC Analysis

**Figure 7:**
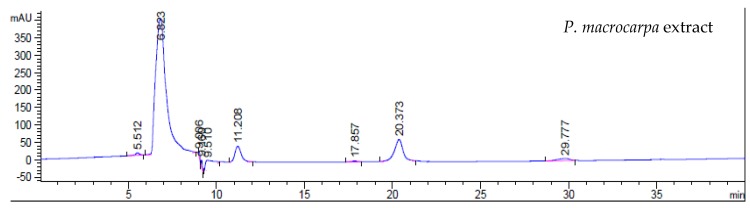

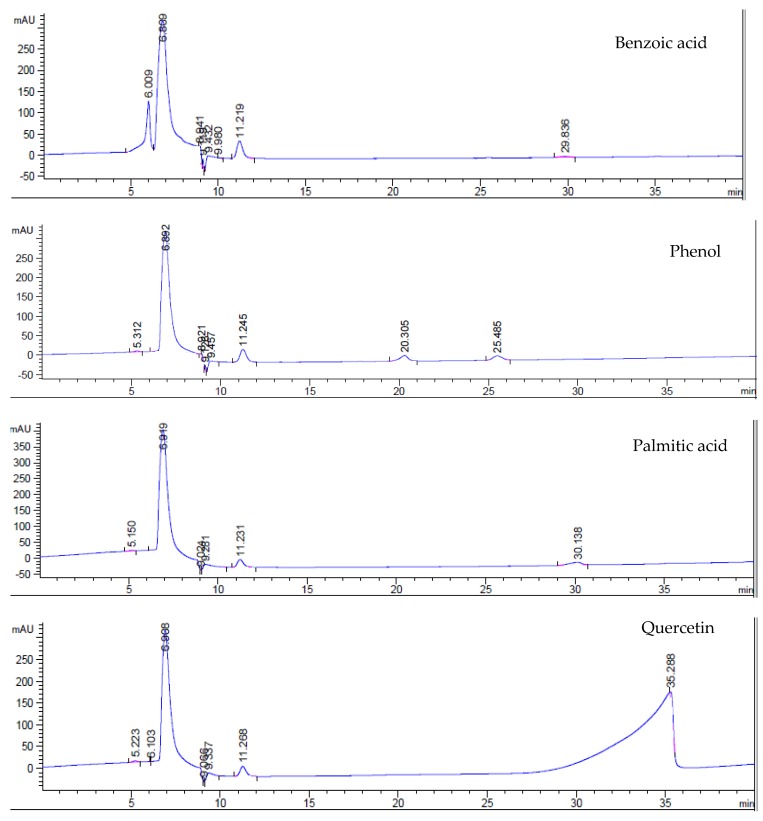
HPLC chromatogram of standards (phenol, benzoic acid, palmitic acid) and *P. macrocarpa* extracts. The extraction used methanol as the extraction solvent.

Extraction yields of *P. macrocarpa* fruit extract was quantitatively determined with high-performance liquid chromatography (HPLC) analysis. Figure 7 shows the HPLC chromatographic profile of the standards (phenol, benzoic acid, palmitic acid, quercetin) and the *P. macrocarpa* fruit extract. 

The value of the sample peak at 6.823 is closer to the peak standards of phenol, benzoic acid, palmitic acid and quercetin at 6.892, 6.809, 6.919 and 6.908. Moreover, quercetin is one of the flavonoid compounds found in *P. macrocarpa* [5]. The peak with retention time at 7.0 min in the *P. macrocarpa* extract agrees well with the retention time of the standard. Thus, the peak at 7.0 min was used to construct a calibration curve and to estimate extraction yields of the found compounds.

### 2.8. Optimization of Extraction Conditions

The main objective of the Response Surface Methodology used in this research was to investigate the levels of experimental factors which gave the highest antioxidant activity in the crude extracts. Four individual verification experiments for free radical scavenging activity (DPPH), ferric ion reducing power assay (FRAP), total phenolic content (TPC) and total flavonoid content (TFC) were carried out under respective optimal extraction time, extraction temperature and extraction solvent to-feed ratios within the experimental range. The final result for the simultaneous optimization using the desirability function approach suggested that the optimal ethanolic extraction conditions for *P. macrocarpa* fruit extract were 66.36 min, 64.10 °C temperature and 74.59% *v*/*v* solvent ratio with desirability of 94%. The value of the missing independent variable in plot was kept at the center point. In order to verify the optimum condition, the *P. macrocarpa* fruit was extracted using the optimal condition above and the results were statistically compared to the predicted values given by the Response Surface Methodological (RSM) model by the Design Expert 7.0.0 software. Based on the results, the predicted values of responses were found to be quite comparable with the experimental values at 94% confidence level (Table 4). This showed that the suitability of the model equation for the prediction of maximum responses was verified using respective responses optimal condition. When constraints in the range were selected, the optimum conditions were obtained. However, it was practically difficult to maintain the conditions during processing and some deviations were expected. Therefore, optimum conditions were targeted as 66 min, 64 °C temperature and 75% *v*/*v* solvent ratio.

## 3. Experimental Section

### 3.1. Plant Material and Chemicals

*Phaleria macrocarpa* was obtained from Skudai in Johor Bahru, Malaysia at latitude 1°32′16.47″ N and longitude 103°39′44.02″ E in November 2015. The red colored fresh fruit was cut into small pieces and dried at room temperature for two weeks. The dried sample was then ground to powder form by using a grinding machine with 0.5 mm mesh size. Ethanol solvent was purchased from Dinamik Sains Sdn.Bhd, (Shah Alam, Selangor, Malaysia) and other chemical reagents used in this study were of analytical grade and purchased from Sigma-Aldrich Malaysia (Subang Jaya, Malaysia).

### 3.2. Extraction Procedure

For the extraction process, about 10 g of dried *P. macrocarpa* fruit was added to 200 mL of ethanol and heated according to the RSM conditions. The mixture was extracted by using a reflux apparatus for extraction times from 66.30 to 133.64 min, temperatures from 53.18 to 80 °C and solvent to-feed ratio at range 63.18 to 96.82 mL/g according to the experimental design with the parameter combination provided by the Central Composite Design (CCD). The solution was then filtered through Whatman No. 1 filter paper to obtain a clear extract. The crude of each extract was collected by using a rotary evaporator (N-N series, EYELA, Tokyo, Japan) at 45 °C and weighed. 

### 3.3. Optimization of P. macrocarpa Extraction Using RSM

#### 3.3.1. Experimental Design

*P. macrocarpa* fruit extraction using ethanol was optimized by employing a Central Composite Design (CCD) with a 2^3^ factorial design consisting of seven factorial points, seven axial points and six central points and 20 experiments. The independent variables in this study were extraction temperature (*X*_1_: 53.18–88.82 °C), time (*X*_2_: 66.36–133.64 min) and ethanol concentrations (*X*_3_: 63.18–96.82% *v*/*v* ethanol/water) which were coded into five levels (−1.682, −1.000, 0.000, +1.000, +1.682). The five levels of values for the independent variables were explicit of their coded and uncoded values as seen in Table 5. The value of independent variables was expressed in their coded values as −1, 0, +1 interval showing the low, medium and high levels of each variable, respectively. The multiple regression analysis was performed on the data of response variables such as DPPH, FRAP, TPC and TFC content as affected by the extraction conditions and was fitted by the response surface regression procedure using the following second-order polynomial Equation (5):

(5) $$Y=\sum A_{0}+\overset{k}{\underset{i=1}{\sum }}A_{ij\;}X_{i}+\overset{k}{\underset{i=1}{\sum }}A_{ij\;}X_{i}^{2}+\;\overset{k-1}{\underset{i=1}{\sum }}\;\overset{k}{\underset{j=i+1}{\sum }}A_{ij\;}X_{i}X_{j}$$

where *Y* is the response variables, *A*_o_, *A_ij_*, *A_ii_* and *A_ij_* are the linear, quadratic and interaction coefficients respectively and *X_i_* and *X_j_* represent the coded values of the *i*th and *j*th independent variables. The variables *X_i_X_j_* represent the first order interaction between *X_j_* and *X_i_* for (*i* < *j*).

#### 3.3.2. Response Surface and Contour Plot

The response surface design was used to evaluate the interaction of three independent variables and their effect on the response. The relationship between independent variables was illustrated by three dimensional responses surface plots by fixing one variables constant at its optimal level [36]. Hence, each of the response surface plots showed the effect of the three selected variables on the antioxidant activities.

#### 3.3.3. Statistical Analysis and Optimization of the Response

Best fitted response models are achieved by displaying statistical parameters such as adjusted multiple correlation coefficients (adjusted *R*^2^, multiple correlation coefficients (*R*^2^), coefficient variation (CV%), lack of fit, regression *F*-value and regression *p*-value by using analysis of variance (ANOVA). Based on analysis of ANOVA, the model obtained is highly significant when (*p* > 0.0001), indicating the good fit of the model [17]. The Prob > *F* test for lack of fit was ensure more than 0.05 (not significant). The *F* test for each individual term in the model was confirmed to be significant. The optimization extraction conditions were selected based on the condition of achieving the highest content antioxidant of DPPH and FRAP, total phenolics and flavonoids by using the desirability function approach in the Design Expert software. The optimum condition of the independent variables was further experimental to obtain the extract and further analysis for cosmetic functional properties. The fitted polynomial equation was expressed in the form of three-dimensional surface plots in order to illustrate the relationship between responses and the experimental used.

#### 3.3.4. Verification of Models

The optimal extraction conditions for DPPH, FRAP, total phenolic and total flavonoid content from *P. macrocarpa* fruit, in terms of extraction temperature, time and ethanol concentrations were determined by correlating the actual experimental values with predicted value from the final response regression equations.

### 3.4. Antioxidant Tests

#### 3.4.1. DPPH Free Radical Scavenging

Anti-oxidant activity of the crude ethanol extract of *P. macrocarpa* fruit was determined by the free-radical scavenging activity, measured by the 1,1-diphenyl-2-picrylhydrazyl (DPPH) method. This method was performed as proposed by Lay et al. [10] with a minor modification. Fifty µL of ethanolic solution of crude extract (10 mg/mL) was placed in a microplate and then mixed with 195 µL of DPPH (50 mg/mL). The mixture was incubated for 30 min in dark conditions at room temperature. After the reaction, the absorbance was recorded at a wavelength of 517 nm on a microplate reader (Spectra Max Plus 384, Molecular Devices Inc., Houstan, TX 77084, USA). This was done by triplicate for each of the crude extracts. The positive control used in this method was ascorbic acid, while ethanol was used as a negative control (blank). The radical scavenging activity of the tested sample was expressed as the inhibition percentage of the DPPH radical scavenging activity, calculated using Equation (6):

% Inhibition = [(*A*_0_ − *A*_1_)/*A*_0_] × 100% (6)

where *A*_0_ is absorbance control and *A*_1_ is absorbance sample

#### 3.4.2. Ferric Reducing-Antioxidant Power (FRAP)

This method measures the ability of antioxidants to reduce ferric iron. It is based on the reduction of the complexity of ferric iron and 2,3,5-triphenyl-1,3,4-triaza-2-azoniacyclopenta-1,4-diene chloride (TPTZ) to the ferrous form at low pH. The FRAP of *P. macrocarpa* fruit extract was determined by using the method of [37]. Firstly, acetate buffer was prepared by mixing sodium acetate trihydrate (3.10 g) with acetic acid (16 mL) and adjusting the volume to 1L using distilled water. Then, TPTZ (0.031 g) was mixed 40 mM HCl (10 mL). Thirdly, ferric chloride (FeCl_3_·H_2_O) solution (0.054 g in 10 mL of distilled water) was prepared. Lastly, FRAP reagent was prepared by mixing 100 mL acetate buffer, 10 mL of TPTZ and 10 mL of ferric chloride solution. The samples were run by mixing 3.0 mL of working FRAP reagent with 100 µL of each crude extract (10 mg/mL). This step was carried out in triplicate and the absorbance was measured immediately at 593 nm using a spectrometer. The procedure was repeated with ascorbic acid as a positive control.

#### 3.4.3. Determination of Total Phenolic Content (TPC)

The total phenolic content of the ethanol extracts was determined by using Folin–Ciocalteu’s reagent adapted to a 96-wells plate assay format with minor modifications [5]. The reaction mixture was prepared by mixing 100 µL of ethanolic crude extract (10 mg/mL), 7.9 mL of distilled water and 2.5 mL of 10% Folin-Ciocalteu’s reagent for 8 seconds in a tube. Then, 2.5 mL of 7.5% sodium carbonate was prepared by dissolving it in distilled water. An amount of 1500 µL of sodium carbonate was mixed into the previous tube solution. The mixture was shaken for 5 min by vortexing and incubated for 2 h at 20 °C in a dark condition at room temperature. Meanwhile, a blank test was prepared containing 100 µL of ethanol, 2.5 mL 10% Folin-Ciocalteu’s reagent dissolved in water and 2.5 mL of NaHCO_3_. The absorption was measured at 765 nm using a spectrophotometer. The sample was prepared in triplicate for each of the crude extract. The same procedures were repeated for the standard solution of gallic acid and a calibration graph of various concentrations was constructed. The phenolic contents (TPC) of the crude extracts were expressed as mg gallic acid equivalent/g of dry weight.

#### 3.4.4. Determination of Total Flavonoid Content (TFC)

The total flavonoid content in each ethanol extract was measured according to Lay et al. [10] with minor modifications. The sample contained 100 µL of ethanol solution of the extract in the concentration of 10 mg/mL and 100 µL of 2% AlCl_3_ solution dissolved in ethanol. The samples were incubated in the dark for 30 min at room temperature and the absorbance was measured at 406 nm. The same procedure was repeated for the standard solution of quercetin and a calibration curve was constructed. Based on the measured absorbance, the concentration of flavonoids (mg/mL) was read from the calibration plot and the content of flavonoid in the extract was expressed in terms of quercetin equivalent (mg of quercetin/g of extract).

### 3.5. Phytochemical Screening of P. macrocarpa Fruit

#### 3.5.1. Fourier Transform Infrared Spectroscopy, FTIR

The analysis was conducted on a 630 FTIR Spectrophotometer System (Agilent Technologies, Santa Clara, CA, USA) at the Department of Chemistry, Faculty of Science, Universiti Putra Malaysia, UPM. A sample of *P. macrocarpa* fruit extract (1.0 mg) was thoroughly mixed with potassium bromide (KBr, 100.0 mg). All the spectra were run over the range from 280 cm^−1^ to 4000 cm^−1^ at room temperature.

#### 3.5.2. High Performance Liquid Chromatography, HPLC

The compounds of *P. macrocarpa* fruit were quantitatively measured by a HPLC technique based on the method described by Crozier et al. [38] with minor modifications. The standards, consisting of benzoic acid, phenol, palmitic acid, and sample extract were analyzed on a series 1200 high-performance liquid chromatography (HPLC) instrument (Agilent) equipped with a UV-Vis photodiode array (DAD) detector, binary pump, vacuum degasser, auto sampler and an analytical column (Nova-Pak^®^ C18 60 Å 4 μm 3.9 × 150 mm, Waters, NANPA, MA, USA). The mobile phase comprised deionized water and acetonitrile. The pH of the water was adjusted to 2.5 with trifluoroacetic acid. The compounds were detected at 365 nm. The column was equilibrated by 100% solvent A (15% acetonitrile) then the ratio of solvent B (35% acetonitrile) was increased to 100% in 20 min. This ratio was maintained for the rest of the analysis with a flow rate of 1 mL/min.

## 4. Conclusions

In this research, Response Surface Methodology (RSM) employing a Central Composite Design (CCD) was successfully used to determine the optimal conditions for the extraction of antioxidant crude extracts from *P. macrocarpa* fruits. The RSM results proved the extraction of phenolic, flavonoid and antioxidant activities are significantly affected by the factors chosen from the preliminary study. The optimal conditions for the nutraceutical extraction of *P. macrocarpa* fruits were found to be extraction temperature at 64 °C, extraction time at 66 min and extraction solvent ratio at 75% *v*/*v*. Under the optimum conditions, the experimental values of DPPH, FRAP, TPC and TFC were in agreement with those predicted, therefore indicating the suitability of the model employed. In future research work, the good optimized result obtained from RSM can be further used for the extraction of *P. macrocarpa* fruit for its cosmeceutical and nutraceutical properties. Hence, RSM definitely provides a numerical structure for dealing with possible discrepancies in the design space. It provides an avenue for efficiently smoothing out numerical noise. It is suggested that the models obtained can be utilized to optimize the extraction time, temperature and solvent ratio for the maximum yield of antioxidant activity.

## Figures and Tables

**Table 1:** The experimental and predicted values for the responses of DPPH, FRAP, TPC and TFC under different concentration.

Run Order ^a^	DPPH ^b^		FRAP ^c^		TPC ^d^		TFC ^e^	
	Exp. ^f^	Pred. ^g^	Exp. ^f^	Pred. ^g^	Exp. ^f^	Pred. ^g^	Exp. ^f^	Pred. ^g^
1	86.70	86.70	7.47	7.47	226.44	226.44	3.22	3.22
2	85.88	85.88	7.29	7.29	212.21	212.20	2.60	2.60
3	84.66	84.68	7.20	7.20	221.64	221.64	2.47	2.47
4	83.70	83.71	7.12	7.12	190.01	190.02	2.32	2.32
5	86.06	86.06	7.21	7.20	242.45	242.47	2.35	2.36
6	86.35	86.35	7.14	7.13	292.33	292.35	1.92	1.92
7	86.25	86.26	7.25	7.28	201.04	201.04	2.11	2.11
8	86.42	86.42	7.30	7.32	233.55	233.55	2.13	2.14
9	86.18	86.18	7.29	7.29	197.95	197.95	2.40	2.43
10	85.60	85.60	7.19	7.17	213.32	213.32	1.93	1.94
11	86.85	86.84	7.31	7.31	292.86	292.88	2.75	2.74
12	84.20	85.20	7.23	7.24	239.38	239.39	2.30	2.30
13	85.41	84.50	7.26	7.26	186.22	186.22	2.90	2.91
14	86.25	86.25	7.19	7.20	236.31	236.30	2.03	2.04
15	85.79	85.79	7.13	7.13	231.20	231.20	2.39	2.39
16	85.79	85.79	7.13	7.13	231.20	231.20	2.39	2.39
17	85.79	85.79	7.13	7.13	231.20	231.20	2.39	2.39
18	85.79	85.79	7.13	7.13	231.20	231.20	2.39	2.39
19	85.79	85.79	7.13	7.13	231.20	231.20	2.39	2.39
20	85.79	85.79	7.13	7.13	231.20	231.20	2.39	2.39

^a^ Run order—randomized; ^b^ Free radical scavenging activity in mg/mL; ^c^ Ferric ion reducing power assay in mg/mL; ^d^ Total phenolic content in (mg/g GAE); ^e^ Total flavonoid content in (mg QE/g of extract); ^f^ Experimental value; ^g^ Predicted value.

**Table 2:** ANOVA for the response surface quadratic model for optimization of DPPH, FRAP, TPC and TFC on the extraction parameters from fruits *P. macrocarpa.*

			DPPH			FRAP			TPC			TFC		
Variance Sources	dF	*p*-Value	Sum of Squares	Mean Square	*F*-value	Sum of Squares	Mean Square	*F*-Value	Sum of Squares	Mean Square	*F*-Value	Sum of Squares	Mean Square	*F*-Value
Model	9	<0.0001	10.98	1.22	17,783.61	0.14	0.016	134.76	14,152.19	1573.58	5.585 × 10^7^	1.84	0.20	5593.88
A	1	<0.0001	0.54	0.54	7811.46	0.013	0.013	108.74	48.05	48.05	1.705 × 10^6^	0.46	0.46	12,619.20
B	1	<0.0001	4.50	4.50	65,592.56	0.055	0.055	465.96	38,891.80	3889.18	1.380 × 10^8^	0.30	0.30	8167.97
C	1	<0.0001	1.80	1.80	26,305.77	0.031	0.031	262.11	5221.77	5221.77	1.853 × 10^8^	0.80	0.80	21,729.86
Interaction														
AB	1	<0.0001	8.450 × 10^−3^	8.45	123.23	3.612 × 10^−3^	3.612 × 10^−3^	30.55	151.12	151.12	5.363 × 10^6^	0.10	0.10	2763.26
AC	1	<0.0001	0.63	0.63	9146.45	6.163 × 10^−3^	6.613 × 10^−3^	55.93	2056.01	2056.01	7.297 × 10^7^	0.014	0.014	394.36
BC	1	<0.0001	2.51	2.51	36,585.80	0.050	0.050	419.62	669.60	669.60	2.376 × 10^7^	0.13	0.13	3549.26
Square														
A^2^	1	<0.0001	0.016	0.016	229.34	0.019	0.032	271.98	1176.92	1176.92	4.177 × 10^7^	0.10	0.10	2769.65
B^2^	1	<0.0001	0.094	0.09	1370.74	0.032	0.014	119.63	2196.13	2196.13	7.794 × 10^7^	0.037	0.037	1000.07
C^2^	1	<0.0001	0.31	0.31	4557.82	0.014	1.182 × 10^−4^		715.94	715.94	2.541 × 10^7^	0.011	0.011	296.27
Residual	10		6.857 × 10^−4^	6.857 × 10^−5^		1.182 × 10^−3^	2.365 × 10^−4^		2.818 × 10^−4^	2.818 × 10^−5^		3.664 × 10^−4^	3.664 × 10^−5^	
Lack of Fit	5		6.857 × 10^−4^	1.371 × 10^−4^		1.182 × 10^−3^	0.000		2.818 × 10^−4^	5.635 × 10^−5^		3.664 × 10^−4^	7.328 × 10^−4^	
Pure Error	5		0.00	0.000		0.000			0.00	0.000		0.00	0.000	
Total	19		10.98			0.14			14,162.19			1.85		
*R*^2^			0.9999			0.9918			1.000			0.9998		
Adj-*R*^2^			0.9999			0.9845			1.000			0.9996		
Pre-*R*^2^			0.9999			0.9313			1.000			0.9985		
Adeq. Pre.			536.669			43.252			28,409.729			302.131		
CV%			9.65			0.15			2.32			0.25		
PRESS			5.269 × 10^−3^			9.926 × 10^−3^			2.316 × 10^−3^			2.781 × 10^−3^		

**Table 3:** FTIR analysis of *P. macrocarpa* ethanol fruit extract.

Functional Group	Absorption (cm^−1^)	Range (cm^−1^)
O-H	3295.68	3400–3200
C-H stretch methylene (asy)	2922.92	2935–2915
C=C-C * aromatic	1613.49	1615–1580
Hetero-oxy compound (nitrogen-oxy, NO)	1273.25	1285–1270
Hetero-oxy compound (silicon-oxy, Si-o-Si)	1041.84	1055–1020

The sign * indicates the presence of benzene ring.

**Table 4:** Optimized level (in the range), optimum level (targeted), predicted optimum value and experimental value of DPPH, FRAP, TPC and TFC.

		**Optimum Value (In Range)**	**Optimum Value (Targeted)**
Variables	Time	66.36 min	66 min
	Temperature	64.10 °C	64 °C
	Solvent to-feed ratio	74.59% (*v*/*v*)	75% (*v*/*v*)
		**Predicted value**	**Experimental value**
Responses	DPPH	86.84%	86.85%
	FRAP	7.47%	7.47%
	TPC	292.88 mg/g	292.86 mg/g
	TFC	3.22 mg/g	3.22 mg/g

**Table 5:** Independent test variables and their coded and uncoded values for CCD matrix.

Variables	Coded and Uncoded Level of Variables				
	−1.682	−1	0	−1	+1.682
Temperature (°C), *X*_1_	53.18	60	70	80	86.82
Time (min), *X*_2_	66.36	90	100	120	133.64
Solvent Ratio Ethanol:Water *v*/*v* (%), *X*_3_	63.18	70	80	90	96.82

## Supplementary Materials

Supplementary File 1
