# Supplementary material for: Development of Phaleria macrocarpa (Scheff.) Boerl Fruits Using Response Surface Methodology Focused on Phenolics, Flavonoids and Antioxidant Properties
Source: Molecules. 2018 Mar 22;23(4):724. doi: 10.3390/molecules23040724 (PMC6017451; doi:10.3390/molecules23040724)
Supplement: Supplementary File 1 [file molecules-23-00724-s001.pdf]

# CERTIFICATE OF PROOFREADING

**THIS IS TO ACKNOWLEDGE THAT THE JOURNAL PAPER ENTITLED**

## DEVELOPMENT OF PHALERIA MACROCARPA (SCHEFF.) BOERL FRUITS USING RESPONSE SURFACE METHODOLOGY ON PHENOLIC, FLAVONOID AND ANTIOXIDANTS

**WRITTEN BY**

**KHURUL AIN**

**HAS BEEN EDITED AND RETURNED TO THE CUSTOMER ON**

**29TH JANUARY 2018**

……………………………………

**INTAN RAIHANA HUSIN**
Chief Executive Officer
MPWS RICH RESOURCES SDN BHD
